# Calcaneous interlocking nail treatment for calcaneous fracture: a multiple center retrospective study

**DOI:** 10.1186/s12891-022-05871-z

**Published:** 2022-10-13

**Authors:** Ye Peng, Junsong Wang, Bo Feng, Yunshou Li, Yunlong Zhu, Weiqing Yuan, Lei Teng, Chengming Zhu, Bin Shi, Lihai Zhang

**Affiliations:** 1 Department of Orthopaedic Surgery, General Hospital of Chinese People’s Liberation Army, Beijing, China; 2 Department of Joint Surgery, Beijing Shijitan Hospital Capital Medical University, Beijing, China; 3 Department of orthopaedic clinical medicine, the Third Affiliated Medical College of Inner Mongolia Medical University, Baotou, China; 4 Department of Hand and Foot Surgery, People’s Hospital of Juxian, Rizhao, China; 5 Department of Orthopaedic Surgery, Beijing fengtai hospital of traditional Chinese and western medicine, Beijing, China; 6 Department of Orthopaedic Surgery, Guangxi Orthopedic Hospital, Nanning, China; 7 Department of Orthopedics, Mayang County People’s Hospital, Huaihua, China; 8 Department of Orthopedics, Liuzhou workers hospital orthopedic/the fourth affiliated hospital of guangxi medical university orthopaedic, Liuzhou, China

**Keywords:** Calcaneal fracture, Sinus tarsi approach, Calcaneal interlocking nail

## Abstract

**Background:**

Minimally invasive treatments for calcaneous fractures have the same outcomes and fewer complications. However, they are technically demanding, and there are a lack reduction tools. To overcome these problems, a calcaneous interlocking nail system was developed that can make reduction and fixation minimally invasive and effective. We retrospectively studied the calcaneous fracture variables intraoperatively and followed up to evaluate the outcomes of patients treated with the calcaneous interlocking nail system.

**Methods:**

All patients in 7 institutions between October 2020 and May 2021 who had calcaneous fractures treated with calcaneous interlocking nails were retrospectively analyzed. The patient characteristics, including age, sex, injury mechanism, Sanders type classification, smoking status, and diabetes were recorded. The calcaneous interlocking nail and standard surgical technique were introduced. The intraoperative variables, including days waiting for surgery, surgery time, blood loss, incision length, and fluoroscopy time, were recorded. The outcomes of complications, AOFAS scores and VAS scores were recorded and compared with other similar studies.

**Results:**

Fifty-nine patients were involved in this study; 54 were male; 5 were female; and they had an average age of 47.5 ± 9.2 years (range 25–70). 2 of these fractures were Sanders type I, 28 of these fractures were Sanders type II, 27 of these fractures were Sanders type III, and 2 of these were Sanders type IV. The surgery time was 131.9 ± 50.5 (30–240) minutes on average. The blood loss was 36.9 ± 41.1 (1-250) ml. The average incision length was 3.5 ± 1.8 (1–8) cm; 57 were sinus tarsi incisions; and 2 were closed fixations without incisions. The average fluoroscopy time was 12.3 ± 3.6 (10–25) seconds during the surgery. The VAS score of patients on the day after surgery was 2.4 ± 0.7 (1–3). The AOFAS ankle-hindfoot score in patients who had a follow-up of at 12 months was 93.3 ± 3.6(85–99). During the follow-up, all patients’ functional outcomes were good. One patient had a superficial infection. The rate of complications of the 59 patients was 1.7% (1/59).

**Conclusion:**

The calcaneous interlocking nail system can have satisfactory reduction and fixation in calcaneous fractures, even in Sanders type IV. The outcomes of follow-up showed good function. The calcaneous interlocking nail could be an alternative method for minimally invasive calcaneous fracture fixation.

## Introduction

Calcaneous fractures are common fractures in the foot [1]. More than 70% of calcaneus fractures are intra-articular fractures [2]. The treatment of displaced calcaneal fractures has been controversial in clinical practice. At present, there are three main treatment methods for calcaneal fractures: conservative treatment, open surgery, and minimally invasive treatment. Because of the reduced soft tissue cover, and the more likelihood of the patient’s smoking, having diabetes and having a high energy injury, open surgery has many complications, such as necrosis of the skin and infection [3–5]. For these reasons, minimally invasive treatments have been popular for calcaneous reduction and fixation. However, minimally invasive treatment is technically demanding and difficult to accomplish for satisfactory reduction and rigid fixation [6–8].

In this study, we try to solve the unsatisfactory percutaneous reduction and rigid fixation problems by calcaneous interlocking nail system. The calcaneous interlocking nail contains reduction tools and fixation system. These reduction tools can reduce the displaced cancellous fracture with traction, and the calcaneous interlocking nail can have rigid fixation of the fracture fragment. The retrospective outcomes of multiple medical centers were reported.

## Materials and methods

### Calcaneous interlocking nail, (Double Medical, China)

**Fig. 1:**
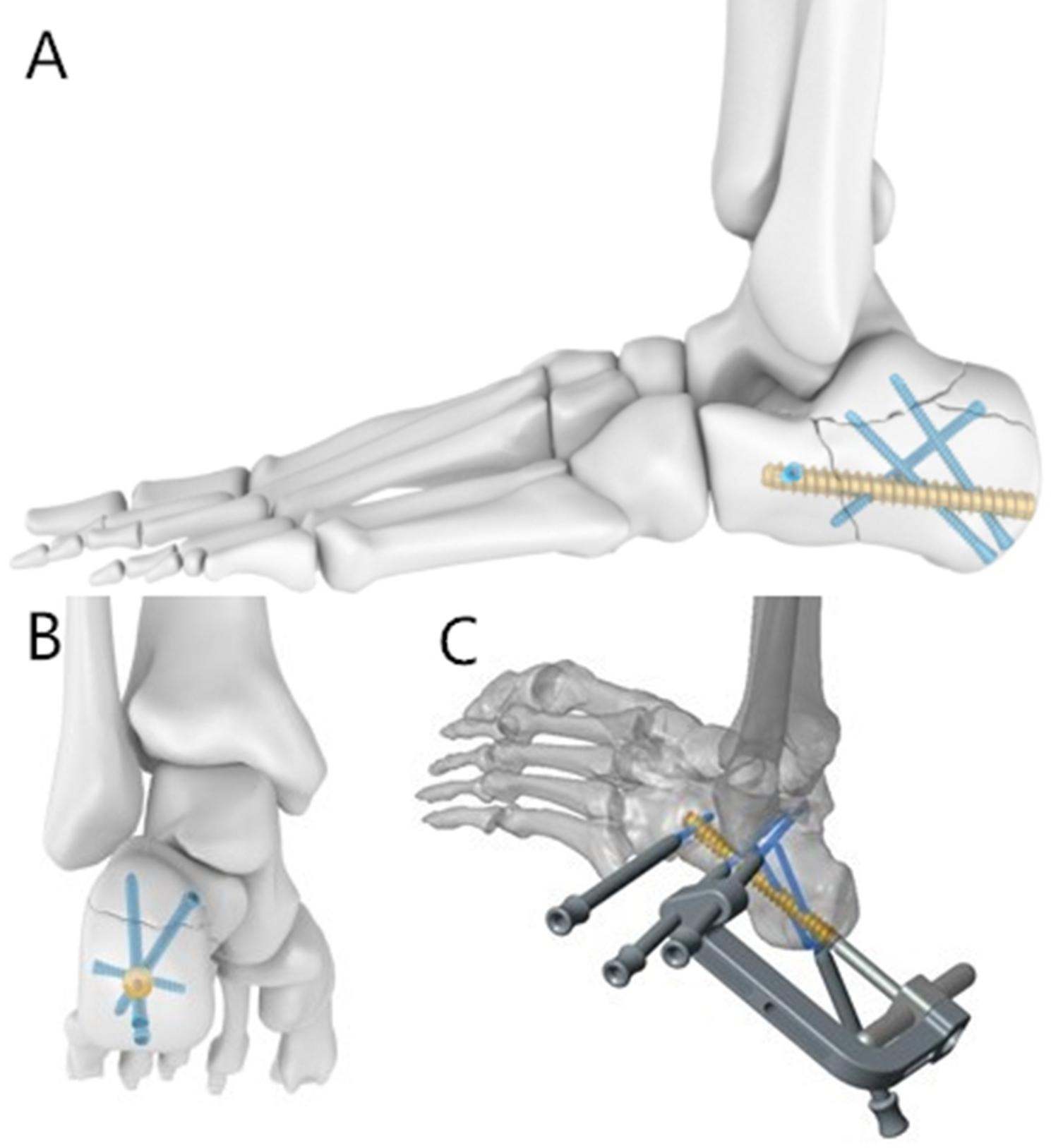
The calcaneous interlocking nail and minimally invasive target device A Lateral view of calcaneous interlocking nail B Posterior view of calcaneous interlocking nail C Target device of calcaneous interlocking nail

The calcaneous interlocking nail is a cannulated titanium alloy nail 65–80 mm long and 8.5 mm in diameter (Fig. 1A, B). The main nail was implanted from the posterior to anterior trough calcaneous tuberosity, which controls the calcaneous length. There are four locking screws for the nail. The anterior lock screw is transverse locking from lateral to medial and controls the rotation of the main nail. The articular fracture fragments have two locking screw fixations that can be implanted from the lower calcaneous tuberosity to the articular fracture fragments to support the fragments. The posterior lock screw is placed from medial superior to lateral inferior and can fix a tongue-type fracture. All locking screws have a target device that can be implanted with minimal invasiveness (Fig. 1C).

### Standard operative technique

**Fig. 2:**
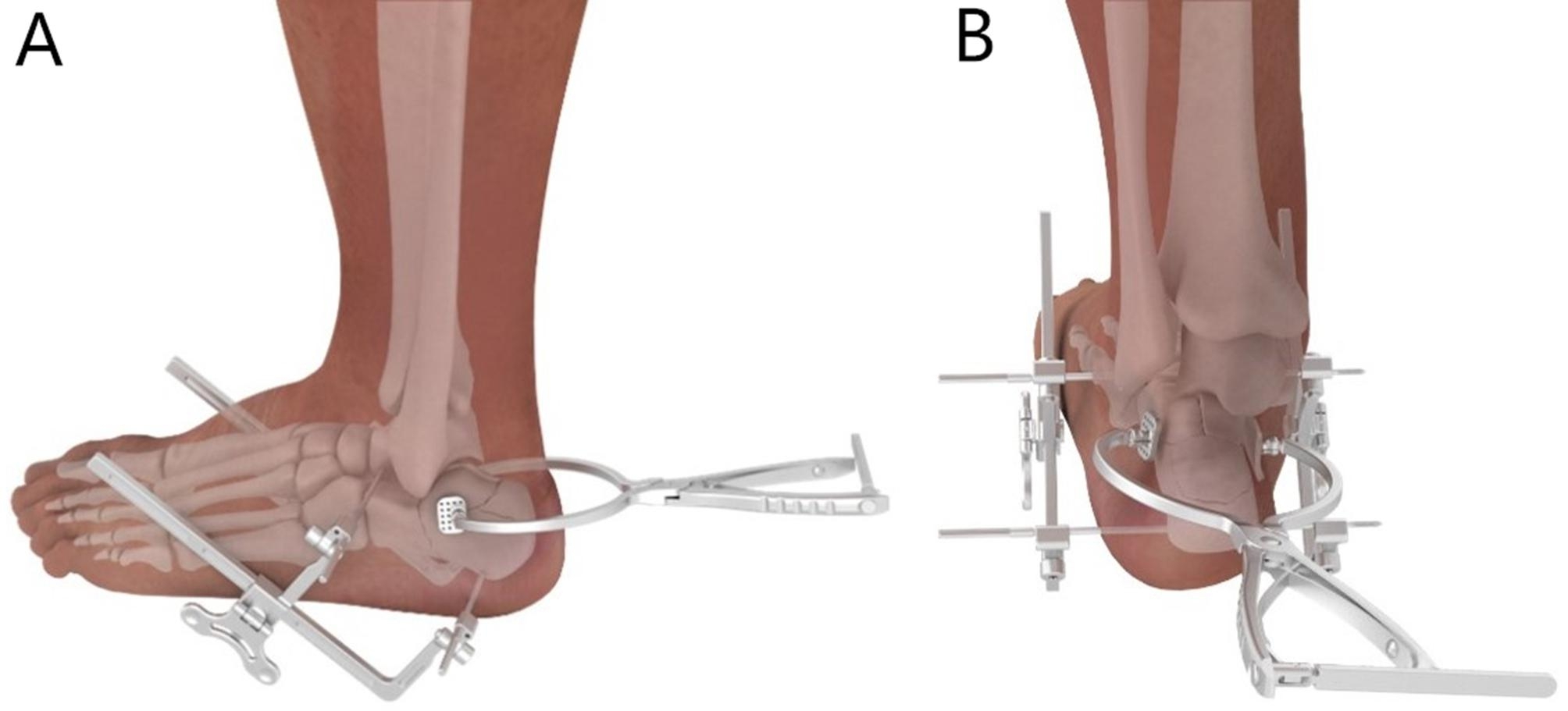
The traction and reduction tools for calcaneous fracture A Lateral view of traction and reduction tools B Posterior view of traction and reduction tools

The patients were positioned in a prone position for percutaneous reduction and interlocking nail percutaneous fixation. The prone position can be used for bilateral surgeries, and a calcaneous axial view is easy to obtain in this position. After routine surgical draping, two 2.5 mm K-wires perpendicular to the talus neck and calcaneus tuberosity were penetrated laterally to medially sequence under a fluoroscope. Then, the two traction devices on both sides of the calcaneus were assembled and were gradually tracted simultaneously to recover the calcaneous shape and create the space for reduction (Fig. 2A, B). The sinus tarsi approach was used, and the subtalar joint and compressed articular fragment were exposed. The compressed articular surface and lateral wall were lifted by a bone elevator. The temporary K-wires were implanted to fix the articular fragment from lateral to medial. For some comminuted fractures, some hollow screws could be implanted for articular surface fixation. The varus and valgus angles of the calcaneus were adjusted by bilateral traction devices under axial view fluoroscopy. The width of the calcaneus was reduced by special-made compression clamps. The calcaneous interlocking nail and four locking screws were implanted in sequence. For severely compressed fractures, bone graft or bone graft substitutes were used to fill the defect space. Finally, the sinus tarsi incision was closed with sutures.

A retrospective analysis was conducted and included 67 patients with displaced intra-articular calcaneal fractures who were treated with calcaneous interlocking nails at 7 institutions between October 2020 and May 2021. The inclusion criteria were patients older than 18 who underwent surgical treatment of a calcaneous closed fracture without other associated fractures. The exclusion criteria were patients who were treated conservatively. Due to the retrospective design of this study, we had no standardized postoperative follow-up protocol, and all the follow-ups were at least 12 months. In total, 59 patients were involved in this study, 54 were male, 5 were female, and they had an average age of 47.5 ± 9.2 years (range 25–70). The injury mechanism, Sanders type classification, smoker, diabetes, days waiting for surgery, surgery time, blood loss, incision length, fluoroscopy time, complications and AOFAS score and VAS score after one year were recorded. X-rays and CT scans were obtained before the surgery: 2 of the fractures were Sanders type I, 28 of the fractures were Sanders type II, 27 were Sanders type III fractures, and 2 were Sanders type IV fractures. The patient characteristics are shown in Table 1.

**Table 1:** Patient characteristics

Age(years)	47.5 ± 9.2(25–70)
Gender	5 female, 54 male
Sanders type classification I II III IV	Number of cases 2 28 27 2
smokers	23/59
diabetes	2/59
days waiting for surgery(days)	7.5 ± 3.2(1–16)

All the patients underwent surgery by surgeons who had more than 5 years of experience with calcaneous fracture fixation. The surgeons were trained for this new calcaneous interlocking nail fixation and reduction. All patients were undergoing surgery according to the standard operative technique.

### Postoperative management

After surgery, full range of ankle motion was permitted the next day, but no weight-bearing was permitted until 4 weeks. During weeks 4–8, patients could start weight-bearing, as tolerated, up to full weight-bearing with a walking stick after surgery. After 8 weeks, the patients were allowed to do some light work without walking sticks. X-rays of the lateral view and axial view were obtained 1 month and 3 months after surgery. All patients were followed up for more than 12 months. The standardized follow-up protocol modified based on Paley and Hall [9] includes radiographic reduction evaluation, incision infection, incision necrosis, peroneal impingement, fracture non-union, fracture malunion, return to work, subtalar joint motion, arthrosis of subtalar and calcaneocuboid joints, footwear problem.

### Statistical analysis

The SPSS statistical software package for Windows (22.0) was used for statistical analysis. Student’s t test was used. Statistical significance was defined as P < 0.05.

### *Clinical outcome* results

The surgery time was 131.9 ± 50.5 (30–240) minutes on average. The blood loss was 36.9 ± 41.1 (1-250) ml. Two patients with Sanders type I underwent closed fixation without an incision. The average incision length was 3.5 ± 1.8 (1–8) cm; 57 were sinus tarsi incisions, and 2 were closed without incisions. The average fluoroscopy time was 12.3 ± 3.6 (10–25) seconds during the surgery. The VAS score of patients on the day after surgery was 2.4 ± 0.7 (1–3). A total of 59 patients reached full weight-bearing, and a calcaneous fracture healing rate of 100% was found by the follow-up. The AOFAS ankle-hindfoot score in patients who had a follow-up of at 12 months was 93.3 ± 3.6(85–99).

**Fig. 3:**
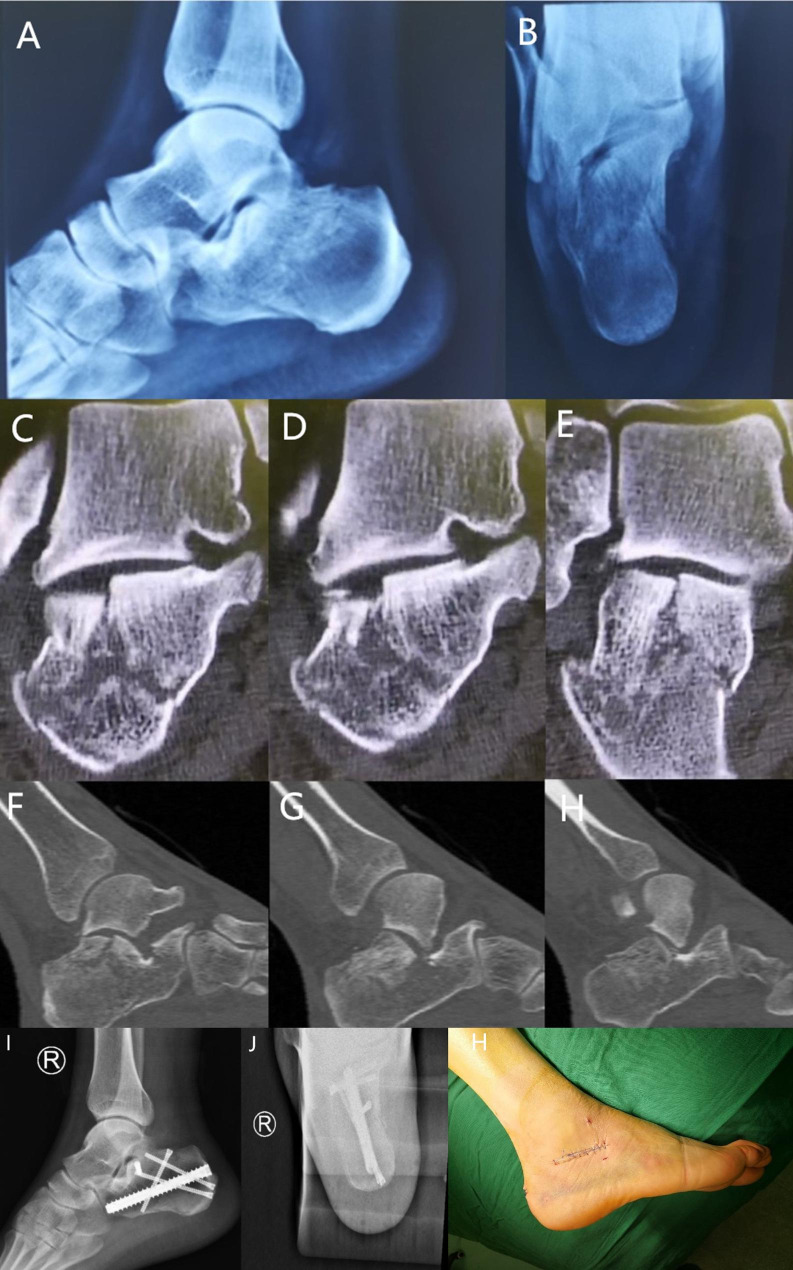
Case 1. Male, 39 years old, who fell from a ladder X-ray showed a calcaneous fracture. Later view (a) Axial view (b)

**Fig. 4:**
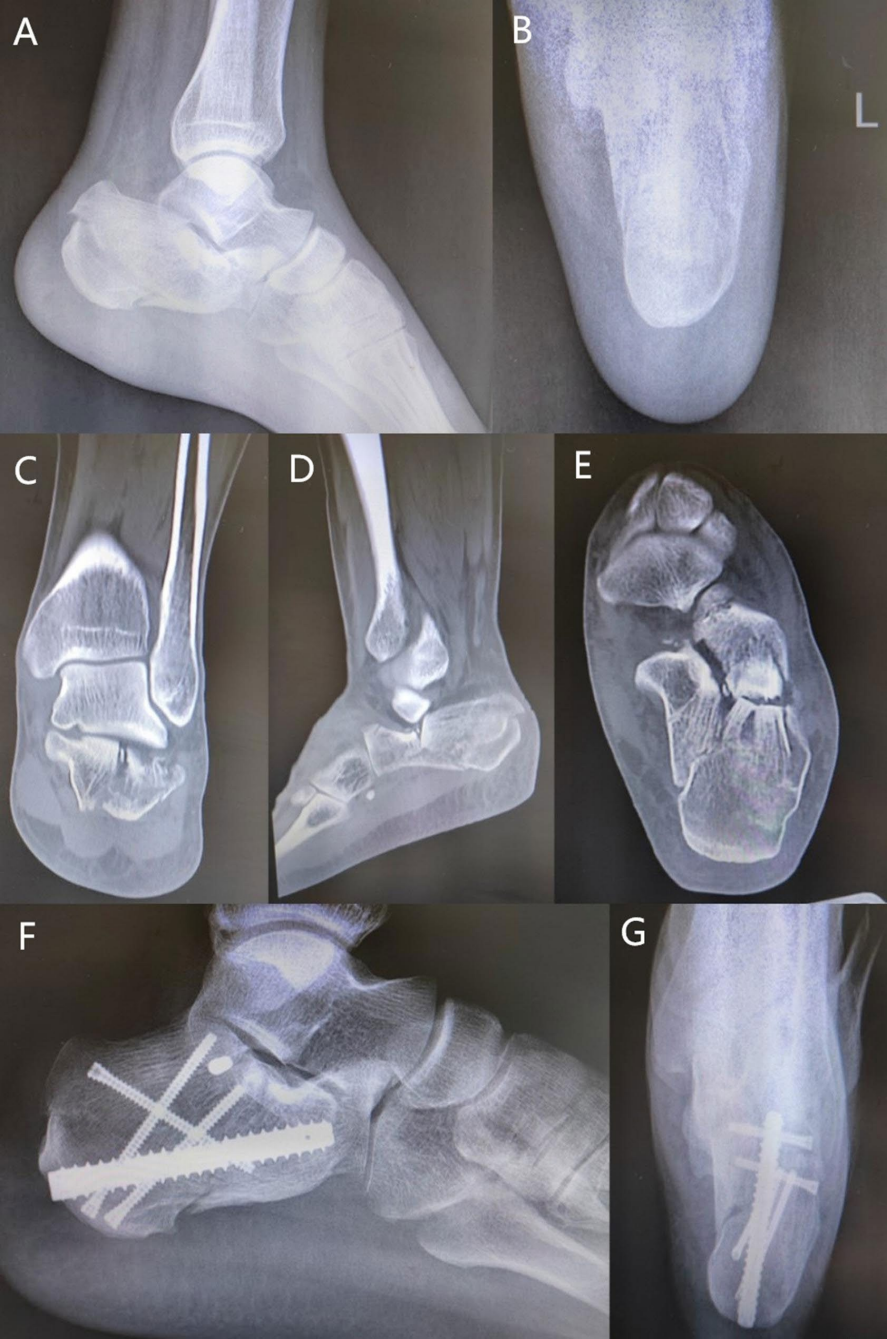
Case 2. Male, 43 years old, who fell from a roof X-ray showed a calcaneous fracture. Later view (a) Axial view (b).

During the follow-up, all patients’ functional outcomes were good. One patient had a superficial infection. The rate of complications of 59 patients was 1.7% (1/59). The patients had dressing changes, and the wounds had healed after one month. No additional surgery was needed. There is no hardware removed. In the follow-up, the radiographic reduction evaluation results showed 89.8%(53/59) anatomic reduction(< 2 mm), 1 patient had incision infection, no patient had incision necrosis, peroneal impingement, fracture non-union, fracture malunion and footwear problems. 54 patients returned to same work and 5 patients changed to light work, average subtalar joint motion 80–95% compared to the opposite one, 1 Sanders type IV patient development arthrosis of subtalar joints. There were some classical cases below (Figs. 3 and 4).

The CT scan showed a depressed fragment with articular steps greater than 2 mm (C-H).

X-ray showed calcaneous fracture reduction and fixation by calcaneous interlocking nail (I-J) sinus tarsi skin incision (3.5 cm).

The CT scan showed a severely depressed articular fragment with widened calcification (C-E).

X-ray showed the calcaneous fracture reduction and fixation by calcaneous interlocking nail (F-G).

**Table 2:** Clinical results of interlock nails for calcaneous fractures

surgery time(mins)	131.9 ± 50.5(30–240)
blood loss(ml)	36.9 ± 41.1(1-250)
incision length(cm)	3.5 ± 1.8(1–8)
fluoroscopy time(s) complications rate AOFAS VAS	12.3 ± 3.6(10–25) 1/59 93.3 ± 3.6(85–99) 2.4 ± 0.7(1–3)

## Discussion

Calcaneous fracture treatment is still controversial. Nonoperatively treated calcaneal fractures are often associated with hindfoot deformities, hindfoot biomechanical disturbances, lateral wall fibular impingement, and peroneal tendon dysfunction. Patients without surgery are 6 times more likely to have their fractures result in late subtalar fusion than patients who had surgery [10]. Open surgery is often associated with the lateral extensile approach, which has great opportunity to cause incision complications, especially with diabetes smokers and high-energy soft tissue injuries. The complication rate of the lateral extensile approach is 20-37% [11–16]. Minimally invasive treatment of calcaneal fractures has gradually become mainstream because it is associated with more rapid healing, lower complication rates, shorter hospital time and less pain [17–19]. To date, many studies have shown that minimally invasive reduction and percutaneous fixation provide the same long-term function and fewer complications [17–19]. However, minimally invasive fixations are technically demanding, and fewer tools and nails have been designed for reduction and fixations. Many minimally invasive methods of calcaneus fixation exist [20–24], but a system that can have easy reduction and fixation is still needed.

For these reasons, we designed a calcaneous interlocking nail system for calcaneous fracture reduction and fixation to overcome the disadvantage of traditionally minimally invasive surgery. The calcaneous interlocking nail system has specially made traction devices, reduction tools and target devices that can make the whole procedure in sequence. Compared to traditionally minimally invasive reduction and fixation, it overcomes the difficulty of free hand reduction, maintains reduction, and implants an accurate screw with the right position under fluoroscopy. The calcaneous interlocking nail system is effective and easy to handle, which lowers the learning curve. All kinds of calcaneous fracture types can be fixed by the calcaneous interlocking system.

A retrospective study from multiple centers showed that 59 patients with calcaneus fractures treated with the calcaneous interlocking nail system had a lower complication rate of 1.6%, which was similar to the 1.9% complication rate of the c-nail [25]. The blood loss was 36.9 ± 41.1 ml using a tourniquet, and most of the blood loss occurred during reduction. The Sanders type I fractures were closed and fixed without an incision. During the reduction, the lateral and axial views of fluoroscopy was used to ensure the articular surface and varus valgus reduction by lateral view and axial view. Many authors use the VAS (visual analog scale) scores and the AOFAS ankle-hindfoot scores to assess the outcomes [26–29]. The next day, the VAS scores were 2.4 ± 0.7, indicating that patients could start rehabilitation exercises as soon as possible. At 12 months of follow-up, the average calcaneous AOFAS ankle-hindfoot score was 65–89. Our study showed that the AOFAS scores were 93.3 ± 3.6, which could be related to very few Sanders type IV fractures. Compared to other studies of calcaneous fractures, Yavuz Akalin [30] reported that 61 patients calcaneous fracture treated with locking plate fixation, the AOFAS average score were 84.7 ± 12.4(t=-5.122 P < 0.001). Wound problems were observed in 15 (28.6%) patients (Z = 3.689, P < 0.001). Eva Steinhausen [31] reported that 33 patients calcaneous fractures treated with C-nail fixation, the AOFAS average score were 80 ± 17 (t= -5.805 P < 0.001). Wound problems were observed in 15 (28.6%) patients (Z = 2.116, P = 0.0034). Takuya Sugimoto [32] reported that 32 patients calcaneous fractures treated with cannulated screw fixation, the AOFAS average score were 90. There was 1 patient had skin necrosis problems in 32 (3%) patients. In summary, the calcaneous interlocking nail for calcaneous fractures showed preferable peri-operation data and lower complications and good functional outcomes during the follow up compared to other studies.

There were some limitations to this study. First, the number of cases was limited, particularly for Sanders type IV fractures. This study is the early experience and applications for calcaneous interlocking nails. Second, the results from reduction, as determined by CT scan, need to be further study. Third, a control group and matched subtype of Sanders classifications should be designed in future studies. Finally, a multicenter randomized comparative study and biomechanical study were not available, which should be performed in the near future.

## Conclusion

This study focused on introducing the calcaneous interlocking nail system and the applications for calcaneous fractures. With the use of this calcaneous interlocking nail system, the surgeons can make minimally invasive reduction and fixation effectively and easily. All procedures can be performed by the target device with minimal invasive incisions. The functional outcome assessments showed that the patients had lower VAS scores and better AOFAS scores after surgery and a lower complication rate. We believe that this calcaneous interlocking nail system could be an alternative method for calcaneous fracture.

## Data Availability

The datasets used and/or analysed during the current study available from the corresponding author on reasonable request.

## Declarations

The treatment was approved from the People’s Liberation Army General Hospital’s Ethics Committee. Informed consent, including publication, was obtained from the patient.
